# In This Issue

**DOI:** 10.1111/cas.14923

**Published:** 2021-05-02

Volume 112, Issue 5, May 2021

## Gastric cancer‐secreted exosomal X26nt increases angiogenesis and vascular permeability

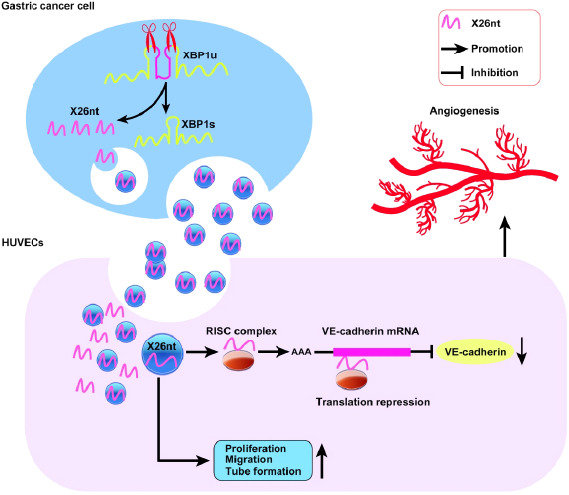

Despite rigorous research in surgery, chemotherapy, and radiotherapy, the prognosis of patients with gastric cancer (GC) has not significantly improved in the last decade. Angiogenesis is one of the hallmarks of cancer and multiples therapeutics have been used for treatment, but our understanding of the mechanisms at play is lacking. In this study, Chen et al investigate the inositol‐requiring enzyme 1 alpha (IRE1α)‐X‐box binding protein 1 (XBP1) pathway and the role that is played in angiogenesis. They focused on the 26‐nt‐long ncRNA (X26nt) that is generated in the process of XBP1 splicing. They noted that X26nt is found in many cancers but was particularly elevated in GC. They discovered that X26nt was delivered to human umbilical vein endothelial cells (HUVECs) via GC cell exosomes and promoted the proliferation, migration and tube formation of the cells. They also showed that its effect on angiogenesis was mediated by targeting vascular endothelial cadherin (VE‐cadherin). This novel mechanism will provide a better understanding of tumor angiogenesis.

https://onlinelibrary.wiley.com/doi/full/10.1111/cas.14740

## Roles of BRCA1 in centrosome regulation and tissue‐specific carcinogenesis

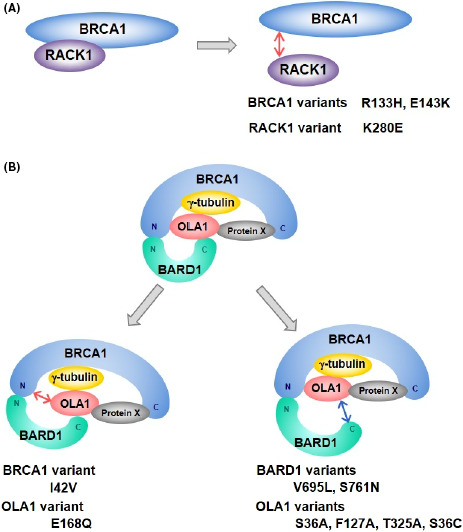

Few genes have as a clear an association with malignancy as breast cancer gene 1 (BRCA1). Deficiency in BRCA1 results in the loss of homologous recombination and thus increases the risk of cancer. BRCA1 is found throughout the body, yet its deficiency or alterations increases the risk of breast and ovarian cancers disproportionately. In this review, Yoshino et al discuss the less studied role of BRCA, centrosome regulation. Centrosomes are microtubule‐organizing centers that are crucial during cell division, and aberrations to centrosome structure or functions have been linked with chromosomal instability and carcinogenesis. Interestingly, deficiencies of BRCA1 and its interacting proteins, such as BRCA1, BARD1, OLA1, and RACK1, result in centrosome aberrations in mammary‐derived cells, but rarely in other cell types. Further investigation into this tissue specific action of BRCA1 may prove fruitful for the treatment of hereditary breast cancer.

https://onlinelibrary.wiley.com/doi/full/10.1111/cas.14859

## GR‐mediated upregulation of GLUT4 in enzalutamide‐resistant prostate cancer

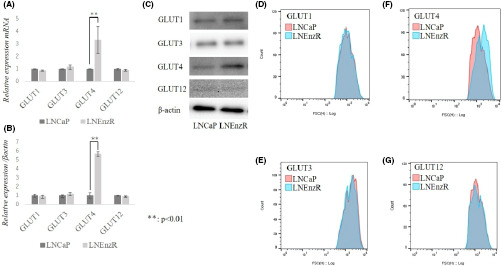

Enzalutamide (Enz) is a small molecule androgen receptor (AR) inhibitor that is reserved for patients with metastatic prostate cancer or castration‐resistant prostate cancer (CRPC). Most patients who are treated with Enz will usually develop resistance. This reality is exacerbated by the fact that there are few efficacious alternatives. In this study, Hoshi et al investigated glucocorticoid receptor (GR) and glucose transporter 4 (GLUT4) and their role in Enz resistance. They developed prostate cancer cells that are resistant to Enz and demonstrated that treatment with Enz increased GR expression in those cells. They also found that GR increased levels of GLUT4 independently of AR. Furthermore, inhibition of GR and GLUT4 reversed the resistance to Enz. More investigation is necessary, but this data provide valuable information about how resistance to Enz develops in CRPC and may have a clinical impact in the near future.

https://onlinelibrary.wiley.com/doi/full/10.1111/cas.14865

